# Stimulating and Recording Brain Signals in an Olfactory Context: Non-Canonical Brain Structures May Contribute to Olfactory Processing – A Case Series

**DOI:** 10.1007/s10548-026-01230-6

**Published:** 2026-07-09

**Authors:** Coralie Mignot, Susanne Weise, Georg Leonhardt, Dino Podlesek, Moustafa Bensafi, Thomas Hummel

**Affiliations:** 1 Department of Otorhinolaryngology, Smell & Taste Clinic, University Hospital Carl Gustav Carus, Technische Universität Dresden, 01307 Dresden, Saxony, Germany; 2 Department of Neurosurgery, ″Technische Universität Dresden″, 01307 Dresden, Saxony Germany; 3 Lyon Neuroscience Research Center, CNRS – INSERM – University Claude Bernard of Lyon, CH Le Vinatier - Bâtiment 462 - Neurocampus, 95 Bd Pinel, Bron, Lyon, 69500 France; 4 Present Address: Department of Neurosurgery, LA‑Regio Kliniken, Klinik Landshut‑Mitte, Robert‑Koch‑Str. 1, 84034 Landshut, Germany

**Keywords:** Intracranial EEG, Stimulation, Stereoelectroencephalography, SEEG, Rhythms, Olfaction

## Abstract

Unlike some other sensory systems, the sense of smell does not benefit yet from a ready-to-use or advanced olfactory implant or prosthesis. The principle of an olfactory implant rests upon volatile compounds that are captured by sensors mimicking the olfactory epithelium, translating them into different stimulation patterns which are then sent to central brain structures. The potential of stimulating a brain structure that does not seem to belong to the primary olfactory pathway, but is part of olfactory networks, has to our knowledge not been considered. We report a case series of patients with pharmacoresistant epilepsy who underwent stereoencephalography (SEEG) for diagnostic reasons, including intracranial stimulation. Firstly, the patients performed a task of passive smelling of two odors (peach and fish), during which electroencephalographic signals were recorded. In a second phase, intracranial electrical stimulation was applied. In two patients, this evoked olfactory sensations specifically after stimulation respectively of the amygdala or temporal pole. Based on these observations, a post-hoc analysis of the SEEG data from the passive smelling task was conducted, focusing on these two structures. Functional connectivity measures have been calculated in different frequency bands using the amygdala or the temporal pole as seeds. Although conclusions are limited due to the small sample size, the results along with the literature underline the interest of considering non-primary olfactory structures, such as the temporal pole, as a contributor to olfactory processing. The efficacy of a potential olfactory implant may rely not only on the primary location of stimulation, but also in the type of oscillations elicited and potential engagement of association brain structures.

**Supplementary Information:**

The online version contains supplementary material available at https://doi.org/10.1007/s10548-026-01230-6.

## Introduction

Worldwide, olfactory dysfunction is common: approximately 20% of individuals have reduced or absent smell, and about 5% are completely anosmic (total loss of smell) (Brämerson et al. 2004; Landis et al. 2004). Although these numbers include alteration of the olfactory system due to normal aging, and depend on the type of olfactory testing (Desiato et al. 2021), they might still slightly underestimate the problem as they were obtained before the SARS-Cov-2 pandemic. Since then, olfactory dysfunction due to viral infections increased and in some cases, remained permanent (Parma et al. 2021; Mitchell et al. 2023; Boscolo-Rizzo et al. 2024). Unlike other sensory systems such as the auditory one with cochlear implants, or the visual one with glasses or retinal implants, the sense of smell does not benefit yet from an advanced olfactory implant or prosthesis, although almost a third of the patients would undergo surgery in the case that they could get such a device (Besser et al. 2019; Stanley et al. 2024).

The principle of an olfactory implant rests upon volatile compounds that are captured by sensors mimicking the olfactory epithelium, translating them into different stimulation patterns which are then sent to central brain structures. The stimulations should be provided via biocompatible electrodes, located most likely in the olfactory system, at the peripheral or the central level. However, many aspects are still not sorted out (Whitcroft et al. 2025). Additionally, recently published data offer promising evidence for the effective and practical application of an olfactory substitution device (Stanley et al. 2025), which is based on an artificial nose coupled to an electrical stimulation system positioned in the nasal cavity that stimulates the intranasal trigeminal system in response to odorants.

In the past, electrical stimulations of the olfactory mucosa of frogs or the olfactory bulb of rats have demonstrated spreading of potentials further in their respective olfactory bulb. This led to a variety of responses in the dorsal field potentials depending of the spatial distribution of electrical stimuli (Ottoson 1959; Coelho and Costanzo 2016; Coelho et al. 2018). In humans, the stimulation of the olfactory epithelium in healthy subjects led to various results, eliciting olfactory percepts in some and none in others (Aronsohn 1884; Uziel 1973; Straschill et al. 1983; Ishimaru et al. 1997; Weiss et al. 2016; Aoyama et al. 2021; Karunanayaka et al. 2023). The olfactory bulb remains the most promising target (Penfield and Jasper 1954; Holbrook et al. 2019; Mignot et al. 2025; Whong et al. 2026) thanks to its surgical accessibility and role in the nature of the olfactory sensation, although further investigation is needed into its precise stimulation site and the human olfactory combinatorial code (Malnic et al. 1999; de March et al. 2020), both of which influence the reproducibility of the elicited sensations. In addition, stimulations of some central structures were successful in eliciting olfactory percepts using deep brain stimulation (DBS) or SEEG in patients with epilepsy, neurological diseases, or obsessive-compulsive disorders. These included stimulations of the olfactory tract (Kumar et al. 2012), the medial orbitofrontal cortex (Bérard et al. 2021), the transverse orbitofrontal sulcus (Fox et al. 2018), the mediodorsal insula (Mazzola et al. 2017), the central sulcus axis of the insula (Li et al. 2023), the amygdala (Andy 1967; Zhang et al. 2023), the thalamus (Nashold and Wilson 1970), and the ventral nucleus accumbens (Okun et al. 2007).

While olfactory processing has been originally studied as a pathway, in broad outline going from the olfactory bulb to the primary olfactory cortex and ending its course in the orbitofrontal cortex (Masaoka et al. 2014), it is recently admitted that this processing is organized in networks. It seems indeed that at least two types of olfactory networks co-exist to be in charge of the pure sensory processing and the refined cognitive one (Karunanayaka et al. 2014; Arnold et al. 2020). Some oscillation patterns have been found to be specifically linked to the olfactory processing (for more discussion on that aspect, see the review (Mignot et al. 2024). Using this knowledge in conjunction with the placement of electrodes might increase the success rate of an olfactory percept under brain stimulation. In that sense, stimulating structures from the primary olfactory cortex appears to be promising to elicit an olfactory percept. To be part of the primary olfactory pathway, the structure of interest usually receives direct inputs from the olfactory bulb via mitral or tufted cells, and has been shown to be essential for basic olfactory perception (and not higher-order odor modulation or association). Beyond that, the potential of stimulating a brain structure that does not seem to belong to this olfactory pathway to all appearances, but is part of these olfactory networks (as they function in synchrony with the primary olfactory cortex), has to our knowledge not been considered.

Here we report a case series of olfactory sensations after intracranial electrical stimulation in patients with pharmacoresistant epilepsy who underwent stereoencephalography (SEEG) for diagnostic reasons, including intracranial stimulation evoking olfactory sensations. SEEG is a technique using depth electrodes to record brain activity or stimulate electrically a localized area of the brain, usually to localize epileptic zones. The patients performed an extra task of passive smelling of two odors (peach and fish), with the resulting EEG signal subsequently analyzed in the amygdala (AMY) and the temporal pole (TP). These two structures were selected based on the initial reports during the experiment about olfactory percepts after electrical stimulation. Functional connectivity measures have been calculated in different frequency bands using the amygdala or the temporal pole as seeds. The amygdala is commonly referred to as part of the primary olfactory cortex (Zald and Pardo 1997; Hudry et al. 2001; Noto et al. 2021), while the temporal pole is not, yet the stimulation of both (separately) led to olfactory percepts in two patients.

## Materials and Methods

### Patients

We recruited patients for our study who fulfilled the following criteria: (i) focal epilepsy proven by scalp EEG, (ii) drug refractory to 3 or more antiseizure medications, (iii) suitable for surgical therapy and (iv) willingness to undergo epilepsy surgery in case it was recommended. The planning of the EEG-investigation that is the placement of the electrodes was exclusively driven by clinical i.e. diagnostic aspects, which is to find the epileptogenic area(s), and our study on olfaction did not have any influence on this aim.

Originally, 9 patients were invited to take part of the study. However, various complications resulted in the non-inclusion of 5 patients. These complications comprised: extensive brain lesions, extensive brain resection from a previous surgery, retraction from participating, intensive seizures with absences, poor cognitive or semantic abilities. The remaining patients’ details are provided in Table 1. Additionally, a technical problem led us to exclude another patient from the passive smelling session (see below). The procedure was approved by the Ethics Committee of the TU Dresden (GVOEK) under the application number BO EK 400,082,021. Patients provided their written informed consent.

### General Procedure

The experiment includes two separated parts: the passive smelling of odors session and the electrical stimulation session.

One or two days before electrodes implantation, a detailed medical history on nasal dysfunction and nasal endoscopy were performed in order to rule out any nasal blockage (polyps, strong septum deviation) or sinonasal disease. Patients were also tested for their olfactory and gustatory performances (see psychophysical tests).

One to five days after electrodes implantation, patients participated to the passive smelling session with two odors presented via a computer-controlled olfactometer. Subsequent SEEG recordings were gathered and analyzed.

On day 8 to 10 after electrodes implantation, and consequently after the passive smelling session, the electrical stimulation session was performed.

After the electrodes were explanted, typically one or two days after the electrical stimulation session, the olfactory and gustatory performances were assessed again for two patients.

### Psychophysical Tests

The psychophysical tests measured the olfactory and taste abilities and lasted around one hour. They were performed at baseline, meaning before electrode implantation, and a second time after the explantation of the electrodes, in order to track these abilities over time and surgery events. This follow-up happened only in two patients, due to the fact that another patient developed a strong headache the day of testing, and for practical reasons for the fourth patient.

The olfactory function was assessed using the Sniffin’ Sticks test (Burghart Messtechnik GmbH, Holm, Germany), a validated tool comprising three subtests: odor detection threshold (T), odor discrimination (D), and odor identification (I) (Hummel et al. 1997). The test employs odor-containing felt-tip pens, which are presented to participants to evaluate their olfactory performance. The detection threshold subtest used an adaptive staircase procedure with a three-alternative forced-choice format, where participants identified a rose-like odor (phenylethyl alcohol) from two blanks. Odor discrimination involved 16 triplets of pens, two containing identical odors and one a different odor, with participants tasked to identify the odd one out. For odor identification, participants were asked to identify 16 common odors from a set of four descriptors each. Scores from each subtest were combined to form a composite TDI score, with normosmia defined as TDI > 30.5, hyposmia as 16.5 < TDI ≤ 30.5, and functional anosmia as TDI ≤ 16.5 (Oleszkiewicz et al. 2019).

Gustatory function was tested using taste sprays of four basic tastes (sweet, salty, sour, bitter) that are sprayed on the tongue (Welge-Luessen et al. 2014). They were randomly presented to the patient, and they had to be recognized twice in order to be considered as correctly assessed.

### Passive Smelling Session

Two odors were used, namely peach and fish aromas (respectively LA-13-00245 and LA-13-00189 from Burghart Messtechnik GmbH, Holm, Germany). Both odors were rated as equally intense in a pre-test. The peach was used at a neat concentration while the fish was diluted 50 times in mineral oil; 20mL of each final solution was placed in a sparger bottle connected to a computer-controlled olfactometer (Sommer et al. 2012). The airflow was set at 2 L/min and odors were provided 20 times each in a mask. At the trial level, each stimulation was delivered for 3 s followed by pseudo-randomized inter-stimulus interval (ISI) duration of 20 s in median. During the ISI, an odorless airflow of equally 2 L/min was provided to the patient. A control condition was added for analyses, consisting in markers placed randomly during the ISI. During the procedure, patients were instructed to look at a fixation cross, and received white noise of approximately 60 dB through headphones in order to mask the clicking sound from the olfactometer. The respiratory signal was measured using a plethysmographic belt, which provides information of changes of tidal volume. The belt was divided into two parts, one placed around the abdomen and the other one on the chest of the patient. The chest respiration was preferred for the analysis.

### Electrical Stimulation Session

Stimulation was applied via a Nihon Khoden 1200 system. Both, low frequency stimulation (1 Hz) and high frequency stimulation (50 Hz) were used at the discretion of the neurophysiologist in charge. The following parameters were used in all stimulations: alternating bipolar rectangular pulses, pulse duration 300 ms, train duration 5–20 s, increasing intensity of 0,2–3 mA (or to 9.8 mA at 1 Hz in one patient), see Table 1 and supplementary material 1 for details. Each stimulation was provided only once. Clinically relevant information (independent from this study) were motor or sensory symptoms or disturbances of language functions (not described here). A sham stimulation (0 mA) was included at each neural site in a random order. For recording intracranial electrodes of Ad-Tech (Ad-Tech Medical, Racine, Wisconsin, USA) (see Table 1) were used. Sampling rate of the EEG was 500 Hz.

Locations of recording were the amygdala (including an area close to the dorsal part of the piriform cortex, and an area close to the ventral part of the insula), the temporal pole (see supplementary material 2), the hippocampus, and the insula. All stimulation sites were selected out of clinical reasons. Thus, some structures not considered as part of the main olfactory pathway were also stimulated, such as the occipital lobe. Yet, it has been shown that such secondary structures can still elicit olfactory percept (Mai et al. 2023). Thus, they still are of interest for the study.

The patients were asked to freely describe whether the stimulation evoked any sensation and which ones they were. Then, they were asked specifically about senses, whether the sensation was an odor, and to describe it in broad words regarding intensity and pleasantness.

### Preprocessing Steps of the EEG Signal

The EEG data were processed with a low pass filter at 235 Hz (FIR filter two-pass, zero phase lag), linear trend and DC offset were removed. Then, they were notch filtered at 50, 100 and 150 Hz. The signal was re-referenced according to the bipolar montage (adjacent contacts). The trials were baseline-corrected via Z-score transformation ((x − µ)/σ where x is the data point value, µ is the mean of the dataset, and σ is the standard deviation) using − 2 to 0 s as a baseline. Epochs were visually inspected and discarded when signs of artifacts or interictal epileptiform activity were found. After that, continuous wavelet transform (CWT) was applied on each trial with a logarithmic frequency definition of 0:50:250 Hz, central frequency of 1 Hz and time resolution (FWHM) of 3s. The subsequent power was baseline-corrected again with Z-score transformation using − 1.8 to − 0.2 s as a baseline interval. Finally, trials were averaged by condition (i.e., peach, fish or odorless) for individual assessments.

### Statistical Analyses

For the electrical stimulation session, we report the patient’s verbal responses to electrical stimulation at a given site.

For the passive smelling session, EEG data analysis was performed with Brainstorm (Tadel et al. 2011) which is documented and freely available for download online under the GNU general public license (http://neuroimage.usc.edu/brainstorm).

Stimulus onsets were manually realigned to the next inspiration phase within the 3 s of odor presentation. The onset marker was placed at the crossover between the inhale curve and the zero line (y axis). Whenever the next inspiration would happen after the end of the odor delivery, the corresponding epoch would be discarded from further analysis.

Pairwise comparisons were made between the odor and the odorless conditions, or between the odorants themselves. They were performed separately for the different frequency bands (theta, beta or gamma) over a time period of 0 to 2.5 s post-stimulus onset. Power values from this time-frequency window were averaged and extracted for each epoch. Number of epochs were equalized between pairs (see Appendices). Non-parametric tests were used as a Shapiro-Wilk test revealed that none of the samples were normally distributed (*p* < 0.05). To compare odor and odorless conditions, a paired one-tailed Wilcoxon signed rank test with 10,000 permutations was performed with further Bonferroni correction controlled over time and frequency dimensions. The reported significance was set at *p* < 0.05.

The lagged coherence was performed across epochs in order to measure the connectivity of two brain structures, taking into account a possible temporal delay, volume conduction and common source artefacts. A Morlet wavelets time-frequency decomposition was used with a logarithmic frequency definition of 1:50:250 Hz, a central frequency of 1 Hz and a time resolution (FWHM) of 3 s. The time-window of interest was 0–2.5 s post-onset. For information, frequency bands are defined as in Pernet et al. (2020): infra-slow: <0.1 Hz, delta: 0.1 to < 4 Hz, theta: 4 to < 8 Hz, alpha: 8 to < 13 Hz, beta: 13–30 Hz, and gamma: >30 Hz. Statistical tests after power and lagged coherence extraction, as well as corresponding figures were performed with JASP: JASP Team (2025). JASP (Version 0.95.3)[Computer software].

## Results

### Electrical Stimulation Session with SEEG

Four patients implanted with SEEG electrodes were included in the electrical stimulation session (Table 1). For the complete table with stimulation parameters, locations, and evoked sensations, see supplementary material 1. The electrical stimulation led to an olfactory percept in two patients. Indeed, in patient #1 a 1 Hz and 1.6 mA stimulation of the right amygdala (AMY) induced a spinach odor perception. In patient #2, an unknown unpleasant odor was perceived while being stimulated at 7 mA and 1 Hz in the temporal pole (TP).

**Table 1:** Overview of the different patients undergoing stimulation during SEEG, the stimulated/recorded structures and the presence or absence of olfactory perception outcome

Patient SEEG #	1	2	3	4
Age	34	39	28	20
Gender	M	M	M	M
Sniffin’ Sticks test (TDI score right and left nostrils) and taste sprays score	Not applicable	Pre-implantation: 37 left nostril and 27.5 right nostril, taste 4/4 Post implantation: 38.5 left nostril and 38.75 right nostril	Pre-implantation: 33, taste 4/4 Post implantation: no follow up test	Pre-implantation: 23 left nostril and 24.5 right nostril, taste 3/4 Post implantation: 29 left nostril and 31.5 right nostril
Site of stimulation and/or recording	Different contacts in the right and left amygdala (AMY)	Contacts in the amygdala (AMY), the hippocampus, right temporal pole (TP), the occipital lobe on the right side, and the right parieto-occipital area	Contacts in the amygdala (AMY), the hippocampus, the insula, the left temporal pole (TP), the frontal lobe and occipital lobe	Not stimulated but recordings from the amygdala (AMY) and the left temporal pole (TP)
Evoked olfactory sensation, stimulation site, stimulation parameter	Spinach odor after stimulation in the primary contact in the right amygdala (AMY) at 1.6 mA, 1 Hz, 5 s	Unknown disgusting odor after stimulation in primary contact in the right temporal pole (TP) at 7 mA, 1 Hz, 5 s.	No olfactory or trigeminal sensations reported	Not stimulated
Comments	Nothing happened during SHAM stimulation	Nothing happened during SHAM stimulation	Nothing happened during SHAM stimulation	Not stimulated

### Passive Smelling Session: Cerebral Olfactory Processing After Odor Delivery

**Fig. 1:**
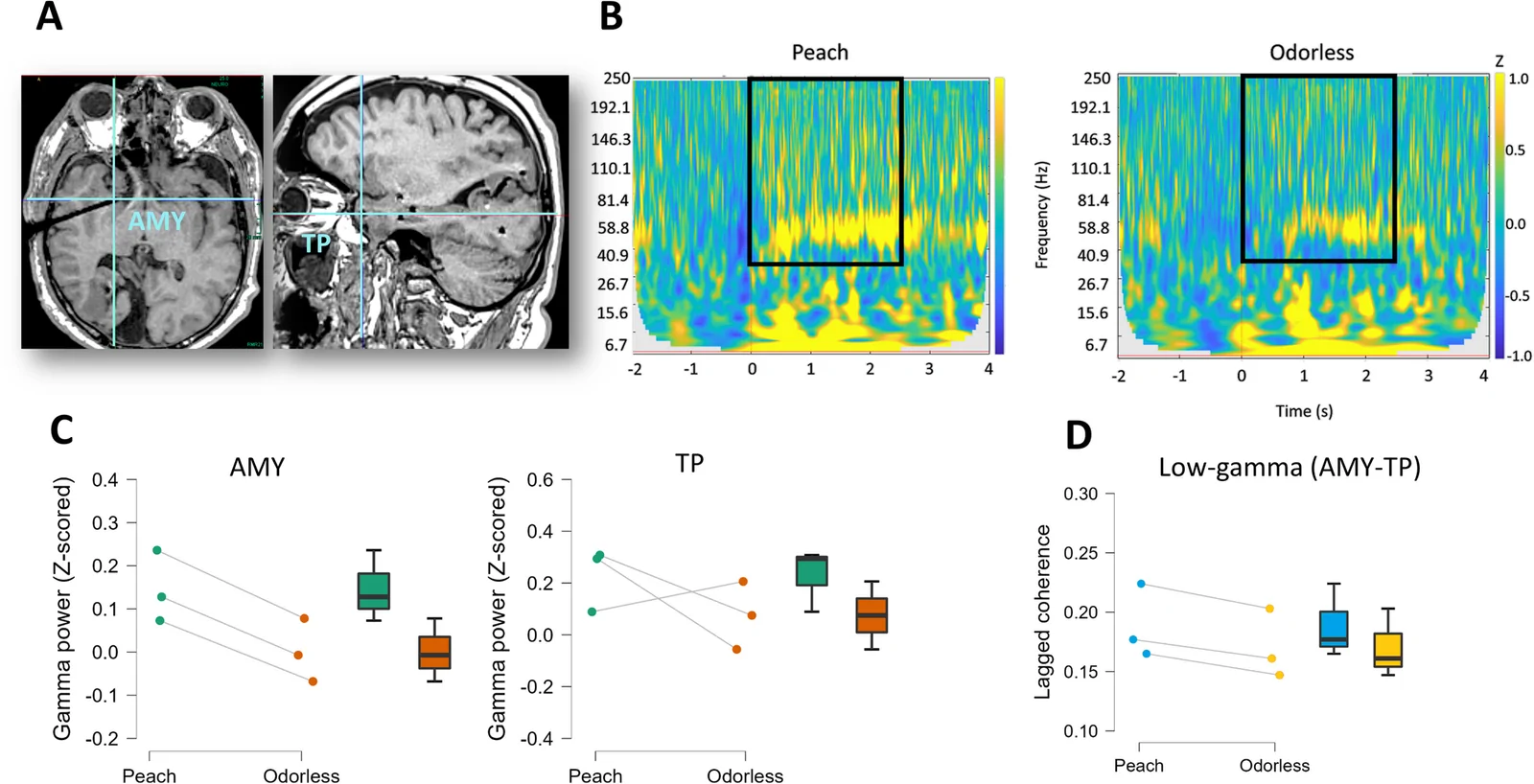
Placement of the electrodes and selection of significant results. (**A**) placement of the electrodes within the amygdala (AMY) and the temporal pole (TP) in patient #2. The blue cross points at the closest contact to the corresponding structure, the electrodes can be observed as black lines going through the skull and reaching the blue cross point. (**B**) Time-frequency analysis of the responses to the peach odor or during an odorless condition in the temporal pole (TP) of patient #2. The power expressed in µV²/Hz was Z scored. The black box underlines the significant difference in the gamma band during the first 2.5s post-onset (Z = 63, *p* = 0.013). (**C**) Increase of mean gamma power in the AMY during peach processing in all three patients as compared to the odorless condition (left graph); increase of mean gamma power in the TP during peach processing in two patients out of three as compared to the odorless condition (right graph). (**D**) Increase of lagged coherence (connectivity) between AMY and TP during peach processing in the low-gamma range for the three patients. AMY: amygdala, TP: temporal pole

The passive smelling of odors session includes data from patients #2, #3 and #4 only, as patient #1 was excluded from that part due to technical problems. Our results from the electrical stimulation of the different brain structures pointed at the amygdala (AMY) and the temporal pole (TP) as potential targets leading to an olfactory percept. Thus, we narrowed down our analyses to these structures in order to understand their patterns of oscillations in an olfactory context (Fig. 1A). The procedure involved an odor of peach, an odor of fish or odorless control conditions delivered via an olfactometer through a constant airflow in the patient’s mask.

#### Psychophysics

A summary of the psychophysics for each patient is given in Table 1. Patient #2 was normosmic from the left nostril and hyposmic from the right one (before implantation T left 11, T right 1.5, D13, I13; after explantation T left 9.5, T right 9.75, D13, I16 – in other words pre-implantation TDI scores were 37 or 27.5 and post-explantation TDI scores were 38.5 or 38.75). No nasal blockage was observed during endoscopy. The taste sensitivity and recognition were normal (score 4/4).

Patient #3 was completely normosmic birhinally before implantation (TDI score 33, with T6, D14, I13) and had a normal sense of taste (score 4/4). Due to a headache, his post-explantation session could not happen.

Patient #4 fell into the hyposmic category before implantation but into the normosmic one after explantation (T left 1, T right 2.25, D11, I11; after explantation T left 2, T right 4.5, D13, I14 – in other words pre-implantation TDI scores were 23 or 24.25 and post-implantation TDI scores were 29 or 31.5). The sense of taste of patient #4 was nearly normal (score 3/4).

The odors were rated by the patients as moderately to highly intense and moderately to highly familiar, except for patient #4. The peach odor was rated as more pleasant than the fish odor. Naming was not accurate but peach was associated to pleasant concepts while fish was associated to more unpleasant ones (Table 2).

**Table 2:** Ratings and description of the odors of peach and fish by the patients

Odor	Patient	Intensity /10	Pleasantness /10	Familiarity /10	Name
Peach	#2	5	7	7	Flowers
	#3	9	10	10	Rose
	#4	2	3	5	Peach
Fish	#2	6	0	5	Gas
	#3	6	3	9	Fish
	#4	2	2	0	Unknown

Ratings were given for intensity (from 0 – absent to 10 – very strong), pleasantness (from 0 – extremely unpleasant to 10 – extremely pleasant, with 5 being neutral) and familiarity (from 0 – completely unknown to 10 – extremely familiar).

#### Brain Oscillations

Pairwise comparisons revealed that gamma oscillations were significantly more present during peach odor processing than during the odorless condition, in primary contacts (targeting the core of the corresponding structure) of AMY and TP for patient #2 (AMY: Power_peach_ = 0.236, Power_control_ = 0.078, Z = 57, *p* = 0.025; TP: Power_peach_ = 0.308, Power_control_ = 0.075, Z = 63, *p* = 0.013, see Fig. 1B). Gamma oscillations were also significantly increased during fish processing compared to the odorless condition in a primary contact in the TP for the same patient (Power_fish_ = 0.119, Power_control_ = 0.029, Z = 44, *p* = 0.047). This patient was the one that reported an unknown disgusting odor after being stimulated in the TP from the same contact. For patient #3, the peach processing involved more beta oscillations at the primary contact in the TP than during the odorless condition (Power_peach_ = 0.843, Power_control_ = 0.442, Z = 26, *p* = 0.040). To access more power values in different frequency bands at the primary contacts in AMY and TP, see supplementary material 2. Other secondary contacts (not targeting the core of the corresponding structure but being further away) showed increased gamma, theta and beta for odor conditions as compared to odorless condition in patients #2, #3 and #4 (see supplementary material 2).

Further connectivity analysis was performed in order to determine whether AMY and TP are functionally connected. Thus, the lagged coherence was calculated while taking both structures as seeds. It was performed separately for each condition (peach, fish, odorless) on a time window of 0–2.5 s post-stimulus onset. Based on qualitative description and trends in a Wilcoxon signed rank test (*p* < 0.125, see Supplementary material 2 for more information), an increase in connectivity was observed for the three patients: in delta during the fish odor processing, in the low-gamma during the peach odor processing (Fig. 1D), as well as in the high-gamma during the fish odor processing.

No apparent correlation or covariation was observed between the different power values or lagged coherences values, and variables such as age, olfactory performances, ratings of corresponding odors, or recognition of such odors. The sample size being small, we cannot exclude such influences.

## Discussion

The present study reports olfactory percepts after electrical brain stimulation of the amygdala (AMY, close to the piriform cortex) and the temporal pole (TP) in two patients out of three.

The involvement of the amygdala in the sense of smell has been discussed extensively. Although very small, it is a hub for different functions and composed of many nuclei (Janak and Tye 2015; Arnold et al. 2020; Noto et al. 2021; Domínguez-Borràs and Vuilleumier 2022; Sakikawa et al. 2023). These are amongst others memory, learning, emotions, olfactory detection and recognition, and valence of odors (Janak and Tye 2015; Patin and Pause 2015; Kulason et al. 2022; Sakikawa et al. 2023). The nuclei are connected to different networks serving different purposes. In humans, the periamygdaloid complex has been hypothesized to participate in the multisensory integration of olfactory information, the medial amygdala would convey the motor responses to olfactory experiences (i.e. fight/flight mode), and the cortical AMY would be involved in olfactory driven reward processing (Noto et al. 2021). In addition, the amygdala has a spatial organization through its direct projections from the olfactory bulb (Noto et al. 2021). In that sense, the olfactory sensation elicited by the stimulation of the AMY can be expected. Other studies have incidentally reported similar results (Andy 1967; Li et al. 2023; Zhang et al. 2023) with stimulation of the amygdala leading to “foul”, “peculiar/obnoxious” or “sour” smells.

On the other hand, the olfactory percept after stimulation in “non primary olfactory structures” such as the TP appears counter-intuitive. Yet, a study from Mai and colleagues (2023) is consistent with the present observations. Authors reported that the stimulation in different brain lobes during radiotherapy in patients with cancer elicited olfactory hallucinations (also known as phantosmias) in 37% of them. Within these patients, 29% of the ones stimulated in the occipital lobe experienced phantosmia, describing most of the time chemical, metallic, or burnt smells (Mai et al. 2023). Whether being a coincidence or not, the description aligns with the one from our patient stimulated in the TP who reported a disgusting unknown smell. Additionally, Fjaeldstad and colleagues found the TP to be anatomically connected with both structural and functional olfactory brain networks, amongst others the piriform cortex, the amygdala, hippocampus, orbitofrontal cortex (Fjaeldstad et al. 2017). Other studies report direct connectivity between the TP and AMY (Chabardès et al. 2002; Sasaki et al. 2023). The TP might be involved in olfactory memory, as its injury causes poor recognition of odors (Rausch et al. 1977; Jones-Gotman and Zatorre 1993). It has also been linked to parosmia, when remaining intact in traumatic brain injury while the olfactory bulb is damaged (Lötsch et al. 2016). While we did not injure the TP during the procedure, these considerations might give insights into why when stimulated in the TP, one of our patients was unable to recognize the odorant and labelled it as disgusting. Indeed, the two olfactory percepts produced here were different in terms of valence and recognition, with the spinach odor described by the patient as a pleasant food item. The TP is a considered as a paralimbic region with dense connectivity to the limbic areas (Sasaki et al. 2023). It has been shown to be involved in the processing of emotionally valenced stimuli including smells (Olson et al., 2007; Royet et al., 2000) and disgust process in the case of visual stimuli (Olson et al., 2007). Thus, stimulating the TP could lead to unpleasant olfactory percepts. However, the amygdala is itself involved in the valence assessment of odorants and its stimulation can also lead to foul smells, as mentioned previously. Thus, the reason behind this valence discrepancy remains open to investigation.

Another point to consider are the differences in stimulation power that produced odor sensation (1.6 mA in the amygdala and 7 mA in the temporal pole). First of all, a given applied current might not be reflected in the same way in two different brain locations, as the effect depends on the tissue conductivity, anatomy, anisotropy, or the presence of surrounding blood vessels and ventricles (Neumann et al. 2023). Thus, it is reasonable that stimulations in the amygdala and the temporal pole might need different current intensities to produce similar oscillations and similar effects (if the oscillation mechanisms needed for olfactory percepts were to be the same). However, there is no certainty that the same oscillation patterns are required in the amygdala and temporal pole to lead to an olfactory sensation. Indeed, it has been shown in other studies during passive smelling that beta and gamma oscillations found in the primary olfactory cortex (including the amygdala) are entrained by theta oscillations (Yang et al. 2022). These theta oscillations are partly a reflection of the smell itself, but also due to the inspiration phase of the breathing cycle (Zelano et al. 2016; Zhou et al. 2021; Yang et al. 2022). The primary olfactory cortex are structures having direct connections with the olfactory bulb. The olfactory bulb might receive information on respiration through the trigeminal collaterals entering it (for work on rodents see Schaefer et al. 2002; Genovese et al. 2017). It is possible, amongst other factors, that due to its lack of direct connection with the olfactory bulb, the temporal pole does not follow the same oscillatory pattern of beta/gamma entrainment by theta than the amygdala. Further studies using frequency-coupling analyses would help in disentangling this point. Secondly, the total electrical energy delivered (TEED) is influenced by the stimulation pulse width, amplitude, frequency and impedance between the electrode and the brain tissue (Neumann et al. 2023). In the case of our two patients, these parameters were identical except for the amplitude and impedance. The latter might have been slightly different from one electrode to another, however we do not believe in our case that this would explain completely the difference of amplitude needed to produce olfactory sensations.

Brain stimulation activates mainly axons, creating antidromic action potentials (back to the corresponding somas) as well as orthodromic action potentials (in the direction of the terminations) (see Brocker and Grill 2013 for review); this means that stimulating the TP could potentiate or inhibit the signal leading to diverse structures, including the ones upstream in the network such as the amygdala, the piriform cortex etc. By stimulating the TP with higher current intensity, we might also have affected the signal from the AMY. This would need more investigation, but remains a possibility. Higher current amplitudes than 1.6 mA in the AMY and 7 mA in the TP did not produce an odor sensation. In fact, higher amplitudes might increase the volume of activated brain tissue (Neumann et al. 2023. As we mentioned, deep brain stimulation usually activates axons leading to downstream and upstream cortical pathways. The effects can vary from activation, suppression or mixed depending on location anatomy and synapses, and electrical parameters (Neumann et al. 2023), which might recruit neurons and networks whose activity is not compatible with the olfactory processing anymore. It is to note that the low frequency stimulation used here (1 Hz) probably have influenced the results as it usually reduces cortical phase synchronization across alpha, beta, gamma and high-gamma, especially if the electrodes are targeting neocortical structures (Manzouri et al. 2021). This effect is generally widespread but more important in the stimulated hemisphere.

Overall, the structural connectivity between the TP and olfactory networks (and the amygdala) could explain why their stimulation may induce olfactory percepts.

We tested in three patients the oscillatory patterns of AMY and TP during a passive odor-delivery experiment involving a pleasant and an unpleasant odorant (peach and fish). We found scattered but significant increases in gamma and beta power in these two brain structures during olfactory processing. A lagged coherence analysis showed that the connectivity between them tended to increase in all patients during smelling in the delta, low-gamma and high-gamma frequency bands, with nuanced results and sometimes non-significant depending on the odor quality. Other work suggested that theta, beta and gamma oscillations increase in the piriform cortex (close to AMY) in response to odors (Jiang et al. 2017; Zhou et al. 2019; Yang et al. 2022; and Mignot et al. 2024 for a review). More specifically, the amount of beta and gamma seems to reflect the odor discrimination and identification performances (Yang et al. 2021). It has been hypothesized that lower frequencies such as theta and beta could serve long-range networking while gamma would reflect local processing (Buzsáki 2006; Thut et al. 2012; Kay 2015; Zhou et al. 2019). Thus, AMY and TP might both have enhanced long-range and local processing during odor perception. The way these oscillations are mimicked or influenced by an artificial input such as electrical stimulation has to be investigated further, as this would constitute the base of an olfactory implant.

While this study together with the literature provides new insights about the interest of considering non-primary olfactory structures, such as the TP, as a contributor to olfactory processing, it is important to acknowledge several limitations. First of all, conclusions are limited due to the small sample size (*n* = 3). Secondly, although a sham stimulation was planned for each stimulation site, and none of them elicited olfactory sensations, a second stimulation would have been valuable in order to prevent false positives or false negatives. Thirdly, another potential bias is the way questions were framed. Just after stimulation, the experimenter started by asking openly if the patient felt any sensation. This was not restricted to olfaction. Afterwards, the experimenter proceeded with bringing attention to different senses including asking specifically about odors. A reason for using such a procedure is that the clinical aim of this session was to locate the focus of epilepsy, thus trying to trigger a seizure. During this process, it is not rare that patients experience a short and transitory amnesic event, and we thought that making them scan their sensations would help them not forgetting to mention anything. In addition, even in the general population, olfaction is considered as the least important of the senses and often forgotten. Fourthly, when applicable, the study would benefit from further questionnaires about the elicited olfactory percept with intensity, pleasantness, familiarity, edibility ratings, frequency of encounters etc. Finally, a further analysis of the respiratory signal at the trial level would be helpful in the future as part of the oscillations in the primary olfactory cortex are entrained by the respiration (Zelano et al. 2016; Yang et al. 2022). We encourage further research to validate our results.

Altogether, we showed that stimulation in the amygdala and the temporal pole can elicit olfactory percepts both neutral or pleasant and unpleasant, and that different oscillations types are increased in a context of passive smelling. By reporting this case-series, we underline that the efficacy of an olfactory implant may rely not only on the location of stimulation in the primary olfactory cortex. We believe that the engagement of association brain structures connected to the primary olfactory cortex could be a potentiation, if not a substitution, for olfactory percepts elicitation. In addition, the type of oscillations provoked by the electrical stimulation may be a key factor, in other words the relationship between electrical stimulation parameters and neurophysiological effects need more investigation. In conclusion, a comprehensive understanding of stimulation targets, parameters and their physiological effects is crucial to pave the way for an olfactory implant, and it may be necessary to map and collect data across larger neural networks.

## Supplementary Information

Below is the link to the electronic supplementary material.

## Data Availability

The data without statistical analysis from one of the patients have been published elsewhere in order to provide context and examples to a review paper (Mignot et al. 2024). The dataset described in the current manuscript is available from the corresponding author on reasonable request.
