# Supplementary material for: Stimulating and Recording Brain Signals in an Olfactory Context: Non-Canonical Brain Structures May Contribute to Olfactory Processing – A Case Series
Source: Brain Topogr. 2026 Jul 9;39(5):76. doi: 10.1007/s10548-026-01230-6 (PMC13350147; doi:10.1007/s10548-026-01230-6)
Supplement: Supplementary file 2 — Supplementary Material 2 [file 10548_2026_1230_MOESM2_ESM.docx]

**
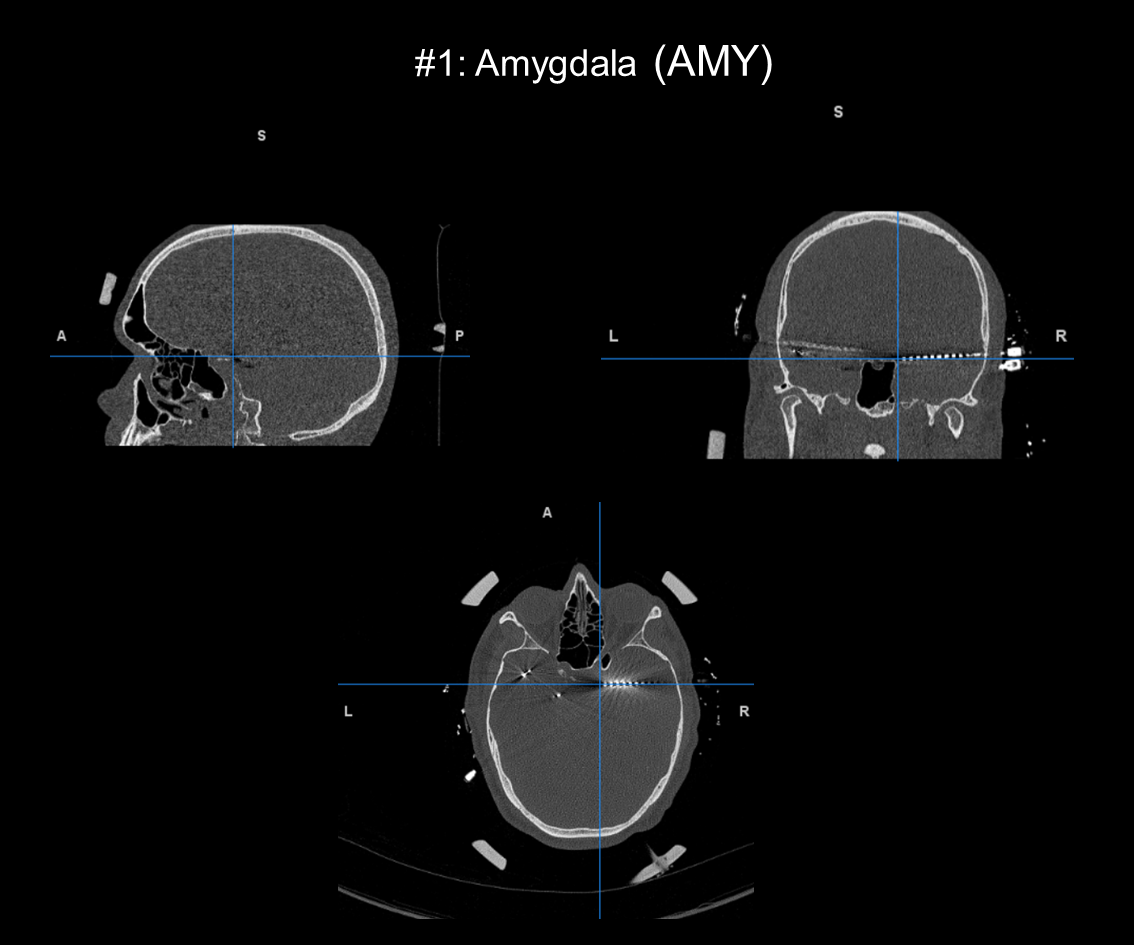
**

Supp. Fig. 1. CT scan of the head from patient #1 showing the electrode targeting the amygdala (blue cross, AMY). For horizontal and coronal views: neurological convention of left and right orientations (left on the left and right on the right).


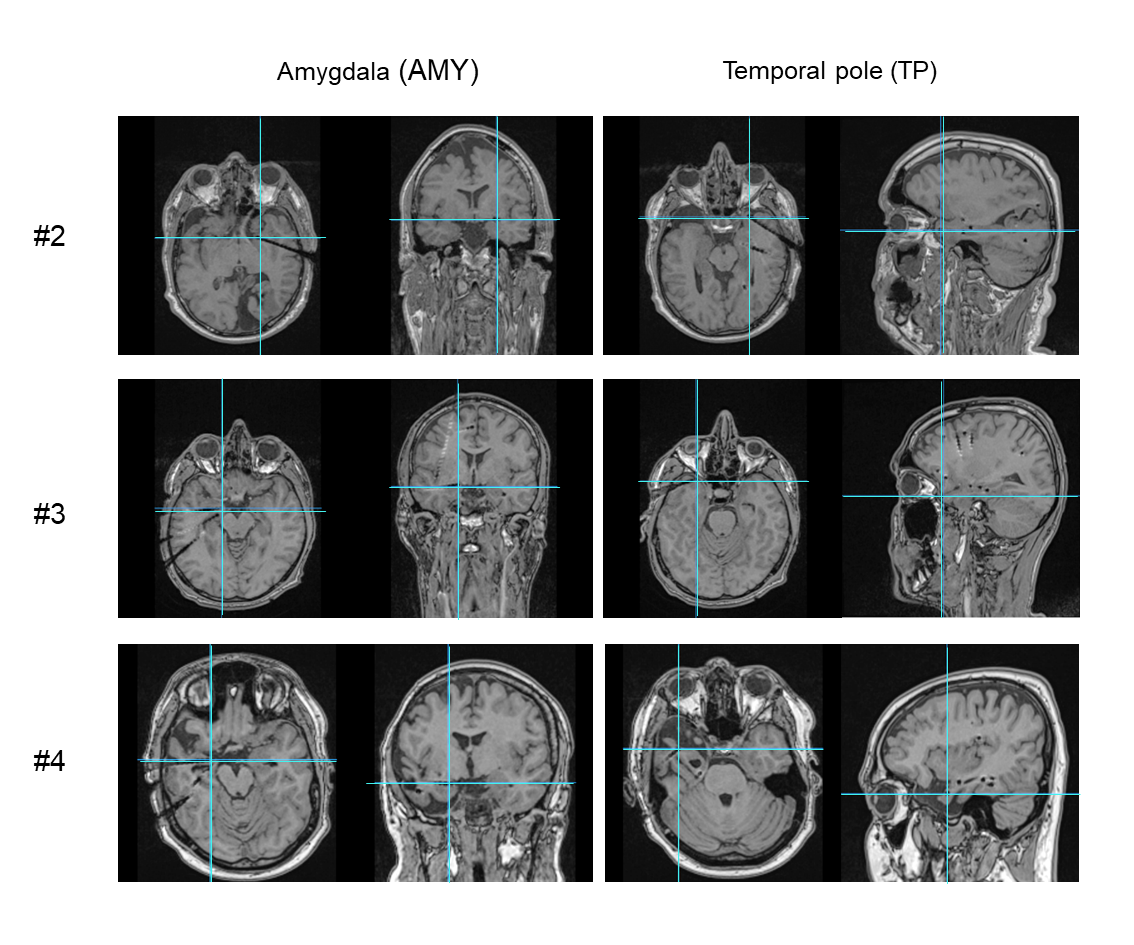


Supp. Fig. 2. Anatomical MRI images of the amygdala (AMY) and temporal pole (TP) of patients #2, #3 and #4. The blue cross indicates the electrode contact targeting the structure of interest. The amygdala is depicted from horizontal and coronal slices while the temporal pole is indicated on horizontal and sagittal slices. For horizontal and coronal views: neurological convention of left and right orientations (left on the left and right on the right).


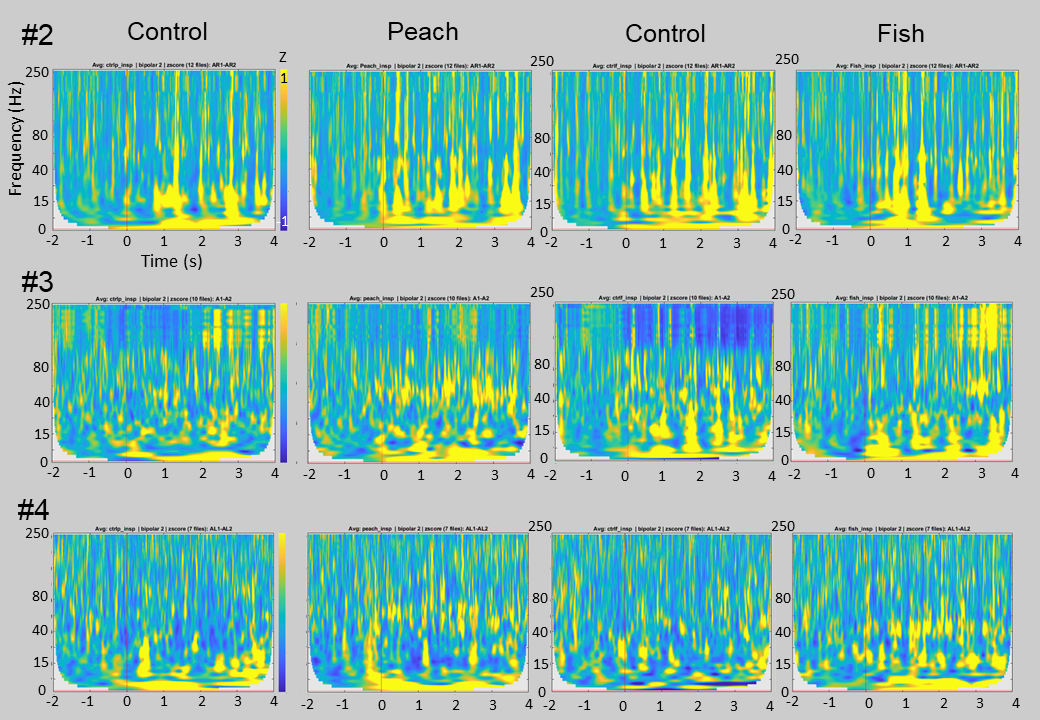


Supp. Fig. 3. Power maps of the responses to peach or fish and their respective control conditions (without odors) from the amygdala (AMY) of patients #2, #3 and #4. The power expressed in µV²/Hz was Z scored. The x axis represents the time (2 s pre-stimulus and 4 s post-stimulus when applicable). Time point 0 was aligned with onset of inspiration phase of the breathing cycle. The y axis represents the frequency from 0 to 250 Hz.


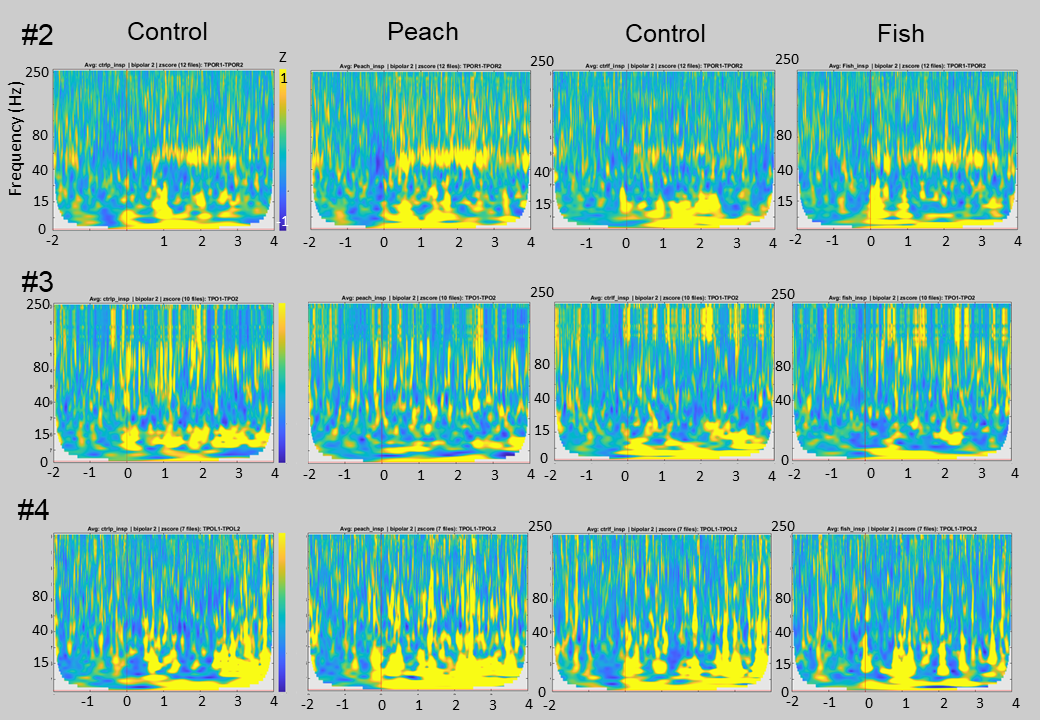


Supp. Fig. 4. Power maps of the responses to peach or fish and their respective control conditions (without odors) from the temporal pole (TP) of patients #2, #3 and #4. The power expressed in µV²/Hz was Z scored. The x axis represents the time (2 s pre-stimulus and 4 s post-stimulus when applicable). Time point 0 was aligned with onset of inspiration phase of the breathing cycle. The y axis represents the frequency from 0 to 250 Hz.

Number of epochs selected for pairwise comparisons and averaged time-frequency maps

Subject 2 – peach vs odorless: 13, fish vs odorless: 12, peach vs fish: 12

Subject 3 – peach vs odorless: 8, fish vs odorless: 7, peach vs fish: 7

Subject 4 – peach vs odorless: 12, fish vs odorless: 10, peach vs fish: 10

Additional pairwise comparisons

| Patient | Comparison | Oscillations (frequency band) | p-value | t-value |
| --- | --- | --- | --- | --- |
| #2 | peach > odorless | Gamma | 0.025 | 57 |

Table supp.1. Summary of significant changes of oscillations across conditions in the amygdala (AMY).

| Patient | Comparison | Oscillations (frequency band) | p-value | t-value |
| --- | --- | --- | --- | --- |
| #2 | peach > odorless | Gamma | 0.013 | 63 |
|  | fish > odorless | Gamma | 0.047 | 44 |
| #4 | peach > odorless | Beta | 0.040 | 26 |

Table supp.2. Summary of significant changes of oscillations across conditions in the temporal pole (TP).
